# The Impact of Osteopontin Gene Variations on Multiple Sclerosis Development and Progression

**DOI:** 10.1155/2012/212893

**Published:** 2012-09-11

**Authors:** Cristoforo Comi, Giuseppe Cappellano, Annalisa Chiocchetti, Elisabetta Orilieri, Sara Buttini, Laura Ghezzi, Daniela Galimberti, Franca Guerini, Nadia Barizzone, Franco Perla, Maurizio Leone, Sandra D'Alfonso, Domenico Caputo, Elio Scarpini, Roberto Cantello, Umberto Dianzani

**Affiliations:** ^1^Interdisciplinary Research Center of Autoimmune Diseases (IRCAD), University of Eastern Piedmont, “Amedeo Avogadro”, Novara, Italy; ^2^Section of Neurology, Department of Translational Medicine, University of Eastern Piedmont, “Amedeo Avogadro”, Novara, Italy; ^3^Department of Health Sciences, University of Eastern Piedmont, “Amedeo Avogadro”, Novara, Italy; ^4^Dino Ferrari Center, The University of Milan, Fondazione Cà Granda, IRCCS Ospedale Haggior Policlinico, Milan, Italy; ^5^Multiple Sclerosis Unit, Don C Gnocchi Foundation, IRCCS, S Maria Nascente, Milan, Italy; ^6^Department of Neurology, Mondovì Hospital, Mondovì, Italy

## Abstract

Osteopontin is a proinflammatory molecule, modulating TH1 and TH17 responses. Several reports suggest its involvement in multiple sclerosis (MS) pathogenesis. We previously reported that OPN gene variations at the 3′ end are a predisposing factor for MS development and evolution. In this paper, we extended our analysis to a gene variation at the 5′ end on the −156G > GG single nucleotide polymorphism (SNP) and replicated our previous findings at the 3′ end on the +1239A > C SNP. We found that only +1239A > C SNP displayed a statistically significant association with MS development, but both +1239A > C and −156G > GG had an influence on MS progression, since patients homozygous for both +1239A and −156GG alleles displayed slower progression of disability and slower switch to secondary progression than those carrying +1239C and/or −156G and those homozygous for +1239A only. Moreover, patients homozygous for +1239A also displayed a significantly lower relapse rate than those carrying +1239C, which is in line with the established role of OPN in MS relapses.

## 1. Introduction

Multiple sclerosis (MS) is an inflammatory disease of the central nervous system characterized by an autoimmune response against the myelin sheaths and axons, resulting in progressive neurological dysfunction [1]. Patients with MS display variable clinical course; at onset, approximately 10% of patients display a primary progressive form (PP), whereas the remainder start out with a relapsing remitting form (RR), and most of them switch to a secondary progressive form (SP) within 10–30 years [2]. Both genetic and environmental factors are involved in the development/progression of MS, and several studies point to a complex inheritance involving interactions between combinations of loci that may influence the immune response [3, 4]. An increasing bulk of data suggest that osteopontin (OPN) may play a role in the pathogenesis of MS [5]. OPN is a 60 kDa-secreted phosphoprotein functioning as a free cytokine in body fluids or as an immobilized extracellular matrix molecule in mineralized tissue [6]. OPN serum levels are increased in several autoimmune diseases and may influence development of these diseases through the OPN immunoregulatory effects enhancing the proinflammatory T helper type 1 (TH1) and TH17 cell responses and inhibiting the TH2 responses [7].

OPN transcript is abundant in plaques dissected from brains of patients with MS, whereas it is absent in control brain tissue; this finding has been confirmed in rat experimental autoimmune encephalomyelitis (EAE) by microarray cDNA analysis of spinal cord tissue [8]. OPN serum levels are higher in relapsing-remitting than in progressive patients, particularly during the relapse [9, 10]. Chowdhury et al. reported a correlation between cerebrospinal fluid (CSF) OPN levels and disease activity in patients with MS. These levels did not correlate with disability status but were higher in patients with active disease [11]. 

The human OPN gene (*OPN*) is located on chromosome 4q22.1, and single nucleotide polymorphisms (SNPs) are associated with development and/or disease activity of several autoimmune diseases [12–14]. A link between the gene and protein data was suggested by the correlation between some* OPN* genotypes and OPN serum levels [15]. Four SNPs of the *OPN* gene (+282T > C in exon VI: rs4754; +750C > T in exon VII: rs11226616; +1083A > G: rs1126772 and +1239A > C: rs9138) in 3′ UTR form three haplotype combinations: haplotype A (282T-750C-1083A-1239A), haplotype B (282C-750T-1083A-1239C), and haplotype C (282C-750T-1083G-1239C). Carriers of haplotype B and C displayed higher OPN serum levels and higher risk of developing autoimmune diseases than haplotype A homozygotes. Several data suggested that the high OPN levels were due to increased stability of the mRNA coded by haplotype B and C [15]. Regarding MS, we previously found that haplotype A homozygotes displayed about 1.5 lower risk of developing MS and lower OPN serum levels than haplotype B or C carriers. Moreover, clinical analysis showed that haplotype A homozygous patients displayed slower switching from a RR to a SP form and milder disease with slower evolution of disability than patients carrying haplotype B or C [16].

Interindividual differences of OPN expression may be also influenced by variations in the promoter region of *OPN* that may modulate its transcriptional activity. This role has been suggested for the −66T > G [17], −156G > GG (rs7687316), and −443>T > C [17] SNPs by Giacopelli et al. [18], and we detected a combined effect of −156G > GG and +1239A > C on risk of systemic lupus erythematosus (SLE) development [14]. 

According to these findings, the aims of this study were (1) to replicate our previous findings on the +1239A>C SNP, (2) to investigate the role of the −156G > GG SNP, (3), to assess the impact of these variations on disease evolution. 

## 2. Materials and Methods

### 2.1. Patients

We analyzed 728 Italian patients (278 males, 450 females; M/F: 0.62) with MS diagnosed according to the revised McDonald criteria [19] and 1218 randomly selected ethnically and age-matched healthy controls. Patients were consecutive patients enrolled from the Multiple Sclerosis Centers of the “Amedeo Avogadro,” University of Eastern Piedmont (Novara), the University of Milan, IRCCS Policlinico Hospital (Milan), the Don C Gnocchi Foundation, IRCCS, S Maria Nascente (Milan), and the “Santa Croce e Carle” Hospital (Cuneo), Italy. Their clinical and demographic features were similar to those of other series [20, 21]. Controls were consecutive Italian donors obtained from the transfusion services of the respective hospitals. Patients and controls were unrelated, Caucasian and Italian, matched for age and gender, with no family history of autoimmune diseases in first degree relatives. According to their clinical course, patients were defined as follows [22]:

RR: occurrence of exacerbations, each lasting at least 24 h and separated by at least one month of inactivity, with full recovery or sequelae (*n* = 447);

PP: steady worsening of symptoms and signs from onset for at least 6 months, whether superimposed with relapses or not, with occasional plateau and temporary minor improvements; (*n* = 71);

SP: initial RR course followed by steady worsening of symptoms and signs for at least 6 months, whether superimposed with relapses or not, with minor remissions, and plateau (*n* = 210). 

We performed an analysis of the following outcome measures: time to reach Kurtzke expanded disability status scale [23] (EDSS) score > 3.0 and time to reach a progressive course, since it was previously shown that OPN SNPs at the 3′ UTR region may influence these measures in MS patients [16]. According to Hawkins and McDonnell [24], disease of patients who, after at least 10 years from onset, had a mild disability, that is, EDSS score ≤ 3.0, was defined benign MS. Patients who reached secondary progression within 10 years from onset were defined fast progressive. Patients who did not reach the endpoints were excluded.

In RR patients, EDSS score was assessed in remission phase. 

The annual relapse rate before treatment was collected in 327 patients with bout onset (RR patients and SP patients) [21]. Only relapses that occurred in the first three years of disease were included in the analysis. 

Samples from patients with RR were drawn during remission. All patients gave their informed consent according to the Declaration of Helsinki [25]. The research was approved by the local ethical committee.

### 2.2. DNA Analysis

Genomic DNA was isolated from peripheral blood mononuclear cells (PBMCs) using standard methods and primers used to evaluate OPN SNPs were the following: 5′-gccgtgaattccacagccatg-3′ (OPN F) 5′-ttgaatgtaataagaatttggtg g-3′(OPN R)(for +1239 SNP) and 5′-agccctctcaagcagtcatc-3′ (promo 1F) 5′-cctgtgttggtggaggatgt-3′ (promo 1R) (for −156 SNP). PCR products were purified with the EXO/SAP kit (GE, Healthcare, Piscataway, NJ, USA). Sequencing was performed with the ABI PRISMR BigDyeTM Terminator kit (Applied Biosystems, Foster City, CA) on an automatic sequencer (Applied Biosystems 3100 Genetic Analyser) according to the manufacturer's instructions.

### 2.3. OPN ELISA Assay

Serum OPN concentrations were evaluated in a capture enzyme-linked immunoadsorbent assay (ELISA) according to the protocol provided by the manufacturer (Calbiochem, San Diego, CA). The optical density was measured at 450 nm with a microplate reader (Bio-Rad, Hercules, CA). The I-smart program was used to create a regression curve. All assays were performed in duplicate, and the observer (E.O.) was blinded to the diagnosis.

### 2.4. Statistical Analysis

Allelic frequencies and outcome measures were compared with the chi-square test with the Yate's correction. Relapse rate was compared with the Mann-Whitney *U*-test. For the ELISA experiments, the approximation of population distribution to normality was tested by using statistics for kurtosis and symmetry. Results were asymmetrically distributed and consequently presented as median values and percentiles. ELISA data comparisons were performed with the nonparametric Mann-Whitney *U* test. All *P* values are 2-tailed and the significance cut-off was *P* < 0.05. 

## 3. Results

We typed the +1239A > C SNP in 728 patients and 1218 controls and the −156G > GG SNP in 728 patients and 912 controls, not overlapping with the cohorts analyzed in our previous study [16]. The +1239A > C SNP was analysed because it allows to discriminate between the A and non-A haplotypes (not carrying versus carrying the +1239C allele, resp.).

Frequency of +1239A homozygotes was decreased in MS patients compared to controls (46% versus 52%; *P* = 0.011), and +1239A homozygotes displayed 1.27 lower risk of MS than +1239C carriers (Table 1). These findings confirmed our previous results on different groups of 425 patients and 688 healthy controls, showing that carriers of the +1239A display a slight protection against MS development. Conversely, no statistically significant difference between patients and controls was found for the −156G>GG SNP (Table 2). 

Genotypic distribution did not deviate significantly from the Hardy-Weinberg equilibrium in any group (data not shown). 

The next step was to assess the impact of these variations on MS evolution, since we previously reported that +1239A homozygotes displayed slower disease progression and milder disability over time compared to +1239C carriers [16]. According to our previous work, disease progression was evaluated by comparing patients switching from RR to SP within 10 years from onset (fast progressive, *n* = 184) and those remaining RR over 10 years (slow progressive, *n* = 444) and disease severity was evaluated by comparing patients with an EDSS score ≤ 3.0 ten years or more after onset (benign MS, *n* = 194) and those who reached a score > 3.0 within ten years (non-benign MS, *n* = 446). 

Table 3 shows that the proportion of slow progressive patients was significantly higher in +1239A homozygotes than in +1239C carriers (80% versus 63%, *P* < 0.0001), whereas no difference was found between −156GG homozygotes and −156G carriers (73% versus 70%, *P* = 0.3). Patients homozygous for both +1239A and −156GG showed a significantly higher proportion of slow progressive patients than those carrying +1239C and/or −156G (95% versus 68%, *P* < 0.0001) and those homozygous for +1239A only (95% versus 80%, *P* = 0.0094). 

Table 3 also shows that the proportion of benign MS patients was significantly higher in +1239A homozygotes than in +1239C carriers (38% versus 24%, *P* = 0.0001) and in −156GG homozygotes than in −156G carriers (46% versus 28%, *P* = 0.0018). Patients homozygous for both +1239A and −156GG showed a significantly higher proportion of benign MS patients than those carrying +1239C and/or −156G and those homozygous for +1239A only (52% versus 38%, *P* = 0.038). 

To further evaluate the clinical impact of *OPN* variations, we analyzed the relapse rate in bout-onset patients carrying different genotypes. Data were available for 327 patients (157 AA, 170 non-AA). The relapse rate was significantly lower in +1239A homozygotes than in +1239C carriers (0.5/yr versus 1.3/yr, *P* = 0.01), whereas no difference was found between −156GG homozygotes and −156G carriers (0.8/yr versus 1.1/yr; *P* = 0.09) or between subjects carrying both protective genotypes and those carrying at least one predisposing genotype (0.6/yr versus 1.2/yr; *P* = 0.06) (Table 4).

Finally, we explored whether OPN serum levels varied in patients displaying different outcomes. Consistently, we found that benign patients, as well as slow progressive patients, showed significantly lower protein levels compared to nonbenign and fast progressive patients, respectively (median value 132 versus 237 ng/mL, interquartile range 94–164 versus 189–289 ng/mL, *P* < 0.0001; median value 154 versus 280 ng/mL, interquartile range 100–207 versus 228–341 ng/mL, *P* = <0.0001).

## 4. Discussion

This work stems from our previous observation of a protective effect of +1239A homozygosity at the 3′UTR of *OPN* for MS development and evolution. In our previous paper, this genotype decreased the risk of MS development by 1.56-fold [16]. The parallel observation of a combined effect of +1239C and −156G on risk of (SLE) development [14] prompted this work extending the *OPN *analysis in MS to −156GG > G. 

The current data, obtained on a much larger independent population, replicated our previous findings on +1239A > C, showing that the frequency of +1239A homozygotes was decreased in MS patients and that these subjects displayed 1.27 lower risk of MS development than +1239C carriers. The same SNPs in the 3′ UTR region of the OPN gene have been studied in 326 Spanish MS patients and 484 controls by other authors. They did not find statistically significant differences between patients and controls, and this apparent discrepancy might be explained by differences in both size and ethnic background of the population under study [26].

By contrast, analysis of −156G > GG SNP did not detect statistically significant differences between patients and controls (OR 0.91, *P* = 0.25), which indicated that this genetic variation was not associated to MS development. To our knowledge, this is the first paper on this SNP in the MS population. 

The most intriguing results were those on the role of these SNPs on the MS course. On the one hand, this study not only confirmed the correlation between +1293A > C and disease progression, but also strengthened this finding showing that +1239A homozygotes displayed a lower relapse rate than the other patients. On the other hand, it detected an additional effect of −156G > GG on disease progression since patients homozygous for both +1239A and −156GG displayed a milder disease, with slower progression of disability and slower switch to secondary progression, than those carrying +1239C and/or −156G and those homozygous for +1239A only. Therefore, −156GG homozigosity in the 5′ end of the gene conferred a further protection especially in subjects also carrying the protective genotype at the 3′ end of the gene.

These protective effects might be related to functional outcomes of these *OPN *variations. In our previous work, in fact, we showed that +1293C was associated with a high “baseline” production of serum OPN, possibly related to increased stability of the OPN mRNA [15]. Moreover, position −156 seems to fall in a putative binding site for a component of the RUNX family of transcription factors and might influence osteopontin expression [18].

A further point supporting a protective role of AA genotype is provided by the analysis of OPN serum levels in patients displaying different disease outcomes. As a matter of fact, patients showing increased frequency of AA genotype, that is, benign and slow progressive MS patients, displayed lower OPN levels. Moreover, our findings are in line with the work by Kariuki SN et al. who reported that *OPN *gene variants modulate cytokine levels in SLE [27].

In conclusion, this work confirms that osteopontin and the *OPN* gene may be involved in MS development and, especially, progression. These observations suggest that this cytokine may be a therapeutic target to counteract MS progression supporting the finding of Steinman et al. showing that anti-OPN antibodies ameliorate the disease course in experimental autoimmune encephalomyelitis [28]. 

## Figures and Tables

**Table 1:** Frequency distribution of OPN +1239A > C genotypes in MS patients and healthy controls.

Genotype SNP + 1239	*MS (*n* = 728)	^†^Controls (*n* = 1218)
AA	335 (46)	634 (52)
AC	314 (43)	486 (40)
CC	79 (11)	98 (8)

AA	335 (46)	634 (52)
Non-AA	393 (54)	584 (48)

	^‡^OR = 1.27 *P* = 0.011 (95% CI: 1.05–1.54)	

*Multiple sclerosis patients.

^†^number of subjects and proportions are shown in the brackets. Genotypic distribution did not deviate significantly from the Hardy-Weinberg equilibrium in any group (data not shown).

^‡^Odds ratio (OR), 95% confidence limits (95% CI), *χ* ^2^ test calculated on allelic frequencies, and *P* values are 2-tailed.

**Table 2:** Frequency distribution of OPN-156G/GG genotypes in MS patients and healthy controls.

Genotype SNP −156	^†^MS (*n* = 728)	^†^Controls (*n* = 912)
GG/GG	78 (10.7)	112 (12.3)
G/GG	304 (41.8)	384 (42.1)
GG	346 (47.5)	416 (45.6)

^‡^OR = 0.91 *P* = 0.25 (95% CI: 0.79–1.06)		

*Multiple sclerosis patients.

^†^Number of subjects, proportions are shown in the brackets. Genotypic distribution did not deviate significantly from the Hardy-Weinberg equilibrium in any group (data not shown).

^‡^Odds ratio (OR), 95% confidence limits (95% CI), *χ* ^2^ test calculated on allelic frequencies, and *P* values are 2-tailed.

**Table 3:** Frequency distribution of different outcomes in MS patients carrying different OPN genotypes.

Outcome	Genotypes					
	+1239A > C		−156GG > G		+1239A > C	
					−156G > GG	
	AA	C	GG/GG	G	AA	C
					GG/GG	G
Fast progressive^b^	57 (20)	127 (37)	18 (27)	166 (30)	3 (5)	181 (32)
Slow progressive^a^	228^c^ (80)	216 (63)	58 (73)	386 (70)	57 (95)	387 (68)
	*P* < 0.0001^d^		*P* = 0.311		*P* < 0.0001	
Benign MS^a^	110 (38)	84 (24)	36 (46)	158 (28)	35 (52)	159 (28)
Non benign MS^b^	183 (62)	263 (76)	42 (54)	404 (72)	32 (48)	414 (72)
	*P* = 0.0001		*P* = 0.0018		*P* = 0.0002	

^a^Patients displaying RR form (slow progressive) or EDSS ≤ 3 (benign MS) after 10 years from onset.

^b^Number of patients displaying that disease status; proportions are shown in brackets.

Patients displaying either RR course and less than 10 years of followup (29/728) or PP course (71/728) were excluded from the analysis of progression. Patients displaying EDSS ≤ 3.0 and less than 10 years of followup (88/728) were excluded from the analysis of disability.

^c^Patients switching to SP form (fast progressive) or reaching EDSS > 3 (non-benign MS) within 10 years from onset.

^d^Statistical analysis was performed by comparing the different outcomes with the *χ* ^2^ test.

Total number in the analysis of progression: 628 patients: 285 AA; 343 non-AA; 76 GG; 552 non-GG; 60 AAGG; 568 non-AAGG.

Total number in the analysis of course 640 patients: 293 AA, 347 non-AA; 78 GG, 562 non-GG; 67 AAGG, 573 non-AAGG.

**Table 4:** Relapse rate in patients with bout onset displaying different OPN genotypes.

Outcome measure	Genotype					
	AA *N* = 153	C *N* = 174	GG/GG *N* = 33	Non-GG *N* = 294	AAGG *N* = 26	CG *N* = 301
Relapse rate	0.5^a^ (0.2–1)	1.3 (0.6–1.7)	0.8 (0.4–1.2)	1.1 (0.5–1.3)	0.6 (0.2–1.3)	1.2 (0.5–1.5)
	P = 0.01^b^		P = 0.09		P = 0.06	

^a^Median values; interquartile ranges are shown in the brackets.

^b^Mann-Withney *U* test.
